# HK2 drives synaptic dysfunction in fragile X syndrome via epigenetic regulation of H3K18 lactylation

**DOI:** 10.3389/fphar.2026.1903604

**Published:** 2026-08-31

**Authors:** Yan Luan, Hanyue Zhang, Ningning Ma, Yuming Zhang, Zhao Xu, Weihua Zhang, Xinyuan Zhang, Yingfei Liu

**Affiliations:** 1 Department of Pathology, Shaanxi Provincial People’s Hospital, Xi’an, Shaanxi, China; 2 Institute of Neurobiology, Xi’an Jiaotong University Health Science Center, Xi’an, China; 3 Xi’an Medical University, Xi’an, Shaanxi, China; 4 Department of Anesthesiology, Shaanxi Provincial People’s Hospital, Xi’an, Shaanxi, China; 5 Department of Hematology, Shaanxi Provincial People’s Hospital, Xi’an, Shaanxi, China

**Keywords:** fragile X syndrome, H3K18la, histone lactylation, HK2, synaptic plasticity, synaptic structure

## Abstract

**Introduction:**

Fragile X syndrome (FXS) is a neurodevelopmental disorder caused by loss of fragile X messenger ribonucleoprotein (FMRP) expression and characterized by synaptic dysfunction and cognitive impairment. However, the metabolic and epigenetic mechanisms underlying these abnormalities remain poorly understood. To address this gap, this study investigated the role of hexokinase 2 (HK2) in FXS pathogenesis and explored downstream metabolic and epigenetic mechanisms associated with synaptic dysfunction and cognitive impairment.

**Methods:**

Using an *Fmr1* ^*−/Y*^ mouse model, we evaluated HK2 expression and its cell-type specificity in hippocampal neurons. RNA immunoprecipitation (RIP) analysis was performed to examine the association between FMRP and *Hk2* mRNA. Genetic knockdown of HK2 was used to assess its effects on neurite outgrowth, synapse density, synaptic transmission, and hippocampal long-term potentiation (LTP). Finally, pharmacological experiments using the HDAC3 inhibitor RGFP966 were performed to examine whether modulation of H3K18la levels contributes to HK2-associated phenotypes.

**Results:**

HK2 expression was markedly elevated in hippocampal neurons *of Fmr1* ^*−/Y*^ mice. RIP analysis revealed an association between FMRP and *Hk2* mRNA, suggesting that HK2 may represent an FMRP-associated transcript. Genetic suppression of HK2 promoted neurite outgrowth, increased synapse density, and improved synaptic transmission and hippocampal long-term potentiation (LTP) in *Fmr1* ^*−/Y*^ mice. Mechanistically, HK2 knockdown selectively reduced H3K18la levels without affecting H3K18 acetylation, supporting a role for HK2 in regulating histone lactylation. Consistent with these findings, pharmacological inhibition of HDAC3 with RGFP966 increased H3K18la levels and attenuated the beneficial effects of HK2 suppression on neuronal morphology, synaptic plasticity, and cognitive function. Behavioral analyses demonstrated that modulation of the HK2-H3K18la axis improved spatial learning and memory in *Fmr1* ^*−/Y*^ mice.

**Conclusion:**

Taken together, these findings support a role for the HK2-H3K18la axis in synaptic dysfunction in FXS. Targeting this metabolic-epigenetic axis may provide a potential avenue for future therapeutic exploration in FXS and related neurodevelopment disorders.

## Graphical Abstract

**Figure:**
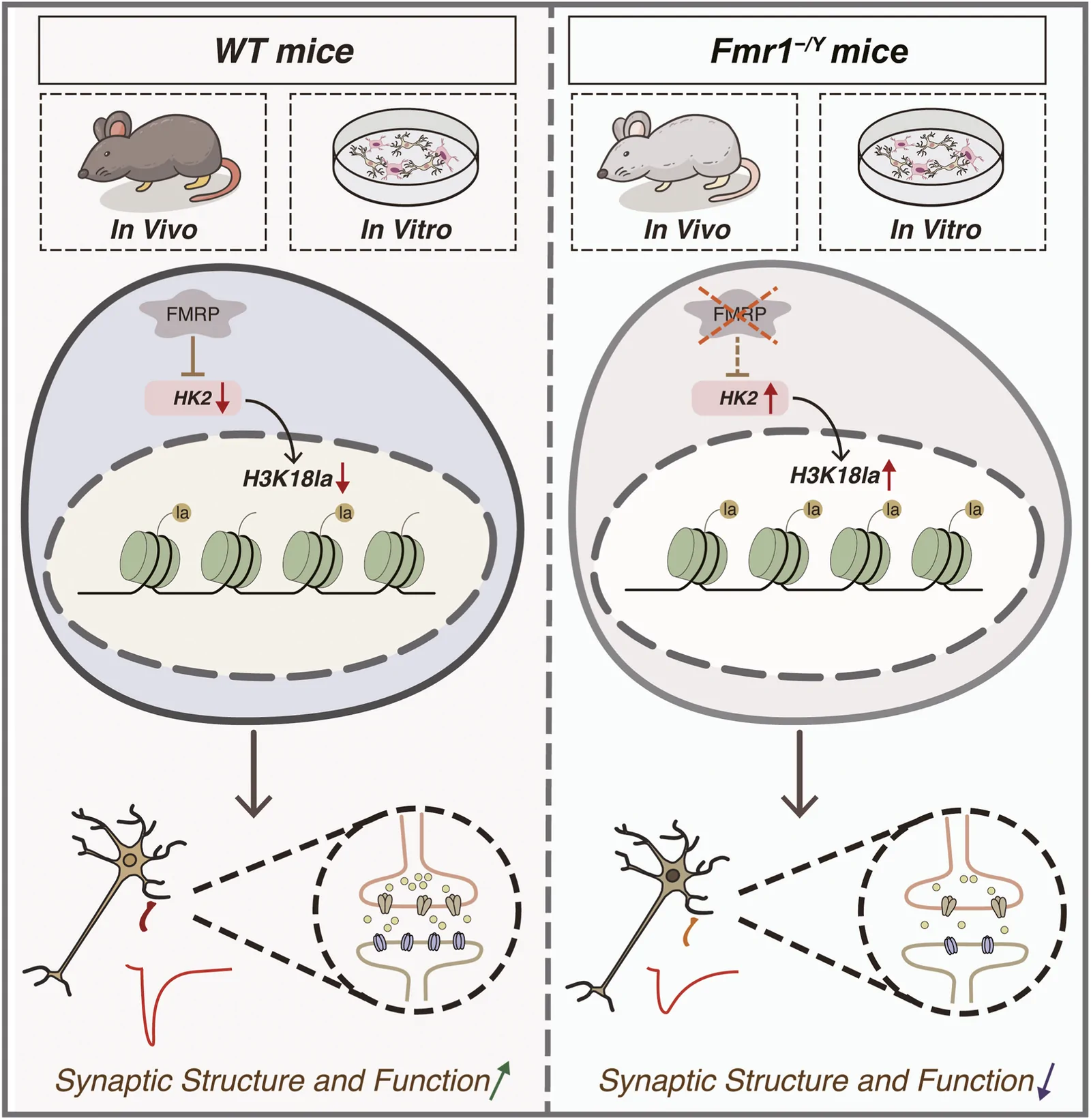
FMRP-HK2-H3K18la positive feedback loop regulates synaptic structure and function.

## Introduction

Fragile X syndrome (FXS) is the most common inherited monogenic cause of intellectual disability and a major genetic contributor to neurodevelopmental disorders. It is caused by CGG trinucleotide repeat expansion in the *FMR1* gene, leading to transcriptional silencing of *FMR1* and reduced or absent expression of fragile X messenger ribonucleoprotein (FMRP) (Verkerk et al., 1991; Genovese and Butler, 2025). As an RNA-binding protein, FMRP plays a critical role in post-transcriptional regulation of neuronal gene expression and synaptic protein homeostasis. Loss of FMRP disrupts mRNA transport, localization, and translational control, contributing to abnormal dendritic spine morphology, altered synaptic architecture, and impaired synaptic plasticity—key features associated with cognitive deficits and autism-related features in FXS (Comery et al., 1997; Roberts and Miyamoto, 2015). Although synaptic dysfunction is recognized as a central pathological feature of FXS, the molecular mechanisms underlying these neuronal abnormalities remain incompletely understood.

Accumulating evidence indicates that mitochondrial dysfunction and metabolic abnormalities are prominent pathological features of FXS (D'Antoni et al., 2020; Mithal and Chandel, 2020). Recent studies have demonstrated that FXS is associated with impaired mitochondrial bioenergetics and altered cellular metabolism, which may contribute to synaptic dysfunction and cognitive impairment (Licznerski et al., 2020; Shah et al., 2026). Consistently, emerging evidence suggests that metabolic remodeling plays an important role in maintaining neuronal homeostasis, synaptic function, and activity-dependent plasticity (Rangaraju et al., 2019; Ashrafi and Ryan, 2017; Magistretti and Allaman, 2018). In addition, metabolic abnormalities have also been reported in individuals carrying the FMR1 premutation; however, these alterations arise from distinct molecular mechanisms compared with those underlying full-mutation FXS (Giulivi et al., 2016). However, how metabolic disturbances contribute to synaptic dysfunction in FXS remains largely unclear.

Among metabolic regulators involved in neuronal energy metabolism, glycolytic enzymes have attracted increasing attention because neurons rely on tightly regulated glucose utilization to support synaptic activity. As a key glycolytic enzyme, hexokinase 2 (HK2) catalyzes the phosphorylation of glucose to glucose-6-phosphate, thereby regulating glycolytic flux and cellular energy metabolism (Afonso et al., 2023; Garcia et al., 2019). Aberrant HK2 expression and activity have been implicated in metabolic dysregulation in various disease contexts (Wu et al., 2022; Liu B. et al., 2023; Ciscato et al., 2021). Beyond its canonical metabolic role, increasing evidence suggests that HK2 contributes to neuronal bioenergetics and mitochondrial function by linking glycolytic activity with oxidative phosphorylation (Bao et al., 2022; Shangguan et al., 2021; Leng et al., 2022; Malard and Mohty, 2020; Zheng et al., 2016). Dysregulated HK2 expression has been implicated in neuronal dysfunction under pathological conditions, and altered HK2 activity and metabolic reprogramming have been associated with neuronal injury and neurodegeneration (Li et al., 2022; Weh et al., 2020; Hu et al., 2022). Collectively, these findings suggest that HK2 represents an important metabolic regulator involved in neuronal physiology and pathological processes. However, whether and how HK2 dysregulation contributes to synaptic dysfunction in FXS remains unclear.

Metabolic alterations, particularly changes in glycolytic flux, can influence epigenetic regulation by altering the availability of metabolic intermediates that serve as substrates for chromatin-modifying enzymes. Beyond these metabolic changes, post-translational histone modifications constitute a fundamental component of epigenetic regulation (Millán-Zambrano et al., 2022). Recent studies have identified multiple histone acylation marks derived from cellular metabolites, including propionylation, butyrylation, 2-hydroxyisobutyrylation, succinylation, malonylation, glutarylation, crotonylation, and β-hydroxybutyrylation (Lin et al., 2022; Liu R. et al., 2023; Taylor and Young, 2021; Wang et al., 2022). More recently, glycolysis-derived lactate has been recognized as a substrate for histone lactylation, a metabolite-derived histone modification that can influence gene transcription (Liberti and Locasale, 2020; Zhang et al., 2019). Histone lactylation has been implicated in diverse biological processes, including macrophage polarization, somatic cell reprogramming, and tumorigenesis, highlighting a connection between cellular metabolism and epigenetic regulation (Yang et al., 2023; Pan et al., 2022; Xie et al., 2022; Yang et al., 2022; Rho et al., 2023). However, existing studies have not fully explored the mechanism of action of histone lactylation in the pathogenesis of neurological disorders, including FXS.

Based on these observations, we hypothesized that HK2 dysregulation may contribute to metabolic and epigenetic alterations in FXS neurons and thereby influence synaptic dysfunction.

## Materials and methods

### Mice

*Fmr1*-KO mice (B6.129P2-Fmr1tm1Cgr/J, RRID: IMSR_JAX:003025, The Jackson Laboratory) were maintained on a C57BL/6J background. Hemizygous male *Fmr1*-KO (*Fmr1* ^*−/Y*^) mice and wild-type (WT, *Fmr1* ^*+/Y*^) littermates at postnatal day 60 (P60) were used in this study. Mice were housed under specific pathogen-free (SPF) conditions in a temperature-controlled environment (22 °C ± 2 °C) with a 12-h light/12-h dark cycle and had *ad libitum* to food and water. All animal procedures were approved by the institutional Animal Care and Use Committee (IACUC) of Shaanxi Provincial People’s Hospital and conducted in accordance with national guidelines for the care and use of laboratory animals. Sample sizes for all experiments are indicated in the corresponding figure legends.

### Primary neuronal culture

Primary hippocampal neurons were isolated from postnatal day 0–1 (P0–P1) male *Fmr1* ^*−/Y*^ mice and wild-type littermates. Hippocampi were dissociated using papain (Worthington Biochemicals), and dissociated neurons were plated onto Matrigel-coated glass coverslips in DMEM/F12 medium (Invitrogen) supplemented with 10% fetal bovine serum (FBS), 2 mM L-glutamine, and 0.1 mg/mL insulin to facilitate initial cell attachment. After 24 h, the plating medium was replaced with Neurobasal medium (Gibco) supplemented with 2% B27 (Gibco). At DIV3, half of the culture medium was replaced with fresh Neurobasal/B27 medium containing cytosine β-D-arabinofuranoside (Ara-C, 2 μM) to inhibit glial proliferation. Cultures were maintained at 37 °C in a humidified incubator with 5% CO_2_ until the indicated time points for subsequent experiments.

### shRNAs and lentiviral transduction

HK2-targeting shRNAs were designed using the Mission shRNA system (Sigma-Aldrich) and cloned into the pLKO.1-puro vector (SHC001, Sigma-Aldrich). All shRNA sequences used in this study are provided in Supplementary Table S1. Lentiviral particles were generated in HEK293T cells by co-transfecting shRNA expression plasmids with the packaging plasmids pRSV-Rev, pMDLg/pRRE, and pVSV-G using polyethyleneimine (PEI). HEK293T cells were transfected at approximately 70%–80% confluence. Viral supernatants were collected 48 h after transfection, clarified by centrifugation to remove cellular debris, and concentrated by ultracentrifugation. The concentrated viral particles were resuspended in sterile phosphate-buffered saline (PBS), aliquoted, and stored at −80 °C until use. Equal volumes of independently prepared lentiviral stocks were used for all experimental groups to ensure comparable transduction conditions. Primary hippocampal neurons were transduced at DIV5 with HK2-targeting shRNA (shHK2) or negative control shRNA (shNC) by directly adding concentrated lentiviral preparations to the culture medium and maintained until the indicated experimental time points. The knockdown efficiency of shHK2 was validated by Western blot analysis in independent cultures before downstream phenotypic analyses. Primary neuronal cultures were randomly assigned to the indicated experimental groups.

### Stereotaxic injection

Lentiviral vectors expressing HK2-targeting shRNA (shHK2) or negative control shRNA (shNC) were stereotaxically injected unilaterally into the right dentate gyrus of P60 mice. Mice were anesthetized with isoflurane and secured in a stereotaxic apparatus. Body temperature was maintained at 37 °C throughout the surgical procedure using a feedback-controlled heating pad. Tapered glass micropipettes (50 μm tip diameter) filled with viral suspension were used for injection into the right dentate gyrus at the following coordinates relative to bregma: AP, −1.94 mm; ML, +1.4 mm; DV, −2.5 mm. Viral suspension (200 nL) was delivered at a rate of 30 nL/min. Micropipettes were maintained in place for an additional 5 min after injection to minimize reflux and facilitate viral diffusion. Mice were randomly assigned to the indicated experimental groups prior to stereotaxic viral injection. Behavioral, electrophysiological, and biochemical analyses were performed 2 weeks after viral injection.

### RGFP966 treatment

For *in vitro* pharmacological inhibition of HDAC3, primary hippocampal neurons were transduced with shHK2 or shNC lentivirus at DIV5, RGFP966, a selective HDAC3 inhibitor (MedChemExpress), was dissolved in dimethyl sulfoxide (DMSO) to prepare a 10 mM stock solution and further diluted in culture medium to a final working concentration of 10 μM. RGFP966 was added to the culture medium at DIV11 and maintained until DIV14, when morphological and electrophysiological analyses were performed. For *in vivo* studies, *Fmr1* ^*−/Y*^ mice were stereotaxically injected with shHK2 lentivirus into the dentate gyrus at 8 weeks of age. Following a 2-week period to allow stable viral expression, mice were treated with RGFP966 (10 mg/kg, i.p.) once daily for 14 consecutive days. Behavioral, electrophysiological, and biochemical assessments were conducted 24 h after the final injection.

### Electrophysiological recordings

Whole-cell patch-clamp recordings were performed on cultured hippocampal neurons maintained on glass coverslips in a recording chamber mounted on an inverted microscope. The extracellular solution contained 145 mM NaCl, 5 mM KCl, 2 mM MgCl2, 3 mM CaCl2, 10 mM HEPES, and 10 mM glucose (pH 7.4; 305 mOsm). Recording electrodes (3–3.5 MΩ) were filled with an internal solution containing 135 mM CsCl, 5 mM NaCl, 4 mM Mg-ATP, 10 mM HEPES, 0.3 mM Na-GTP, 5 mM EGTA, and 5 mM QX-314 bromide (pH 7.2; 300 mOsm). Signals were acquired using an Axopatch 700B amplifier connected to a Digidata 1440A interface and analyzed using Clampfit 11.1 software. Series resistance was continuously monitored and compensated by >80% throughout recordings. Recordings were excluded if series resistance exceeded 25 MΩ or changed by more than 20% during the experiment.

Miniature excitatory postsynaptic currents (mEPSCs) were recorded at a holding potential of −70 mV in the presence of tetrodotoxin (TTX, 0.5 μM).

For hippocampal long-term potentiation (LTP) recordings, acute hippocampal slices were transferred to a Med64 microelectrode chamber and continuously perfused with oxygenated artificial cerebrospinal fluid (95% O2/5% CO2; 2 mL/min) at room temperature. Field excitatory postsynaptic potentials (fEPSPs) were recorded in the dentate gyrus (DG) following stimulation of the perforant pathway. Stimulation intensity was adjusted to evoke 30%–40% of the maximal fEPSP response. Test pulses were delivered at 0.05 Hz, and a stable baseline was recorded for at least 20 min before LTP induction. LTP was induced using theta-burst stimulation (TBS) consisting of 5 bursts delivered at 5 Hz, with each burst containing 4 pulses at 100 Hz. Following TBS, fEPSP responses were recorded for 180 min. The slopes of fEPSPs were normalized to the average baseline values for analysis.

### Immunostaining

Brain sections and cultured neurons from different experimental groups were fixed in 4% paraformaldehyde (PFA) for 15 min and blocked with 5% bovine serum albumin (BSA; Sigma-Aldrich) for 2 h at room temperature. Samples were then incubated overnight at 4 °C with primary antibodies, followed by three washes with 0.01 M phosphate-buffered saline (PBS) and incubation with appropriate fluorophore-conjugated secondary antibodies (Invitrogen, USA) for 2 h at room temperature. Nuclei were counterstained with DAPI (H-1200, Vector Laboratories, USA). Images were acquired using a BX51 fluorescence microscope equipped with a DP70 digital camera (Olympus). All antibodies used in this study are listed in Supplementary Table S1.

Dendritic complexity was quantified using Sholl analysis in ImageJ software. Concentric circles were centered on the neuronal Soma at 20-μm intervals, and the number of dendritic intersections at each radius was quantified. Fifteen neurons from three independent cultures were analyzed per experimental group.

### RNA immunoprecipitation (RIP)

RNA immunoprecipitation (RIP) was performed to examine the association between FMRP and Hk2 mRNA. Hippocampal tissues were lysed in RIP lysis buffer (25 mM Tris-HCl, pH 7.4, 150 mM NaCl, 1% NP-40, 0.5% sodium deoxycholate, and 0.1% SDS) freshly supplemented with Complete Protease Inhibitor Cocktail (Roche) and RNase inhibitor (Invitrogen, 40 U/mL). Lysates were clarified by centrifugation at 12,000 × g for 15 min at 4 °C, and protein concentrations were determined using a BCA protein assay.

For each RIP reaction, 500 μg of total protein lysate was precleared with Protein A/G PLUS-Agarose beads (Santa Cruz Biotechnology, sc-2003) for 1 h at 4 °C to reduce non-specific binding. Prior to use, the beads were pre-blocked with 1 mg/mL yeast tRNA and 1% bovine serum albumin (BSA) for 2 h at 4 °C. Precleared lysates were then incubated overnight at 4 °C with an anti-FMRP antibody or normal rabbit IgG as a negative antibody control (Supplementary Table S1). Subsequently, 50 μL of pre-blocked Protein A/G PLUS-Agarose beads were added to each sample and incubated for an additional 2 h at 4 °C. The RIP assay was performed under native (non-crosslinked) conditions to preserve endogenous RNA–protein interactions. Immunoprecipitates were washed five times with RIP wash buffer (50 mM Tris-HCl, pH 7.4, 300 mM NaCl, 1 mM MgCl_2_, and 0.05% NP-40) supplemented with protease and RNase inhibitors. Following the final wash, approximately 10% of each immunoprecipitated sample was eluted in Laemmli sample buffer and analyzed by Western blot using an anti-FMRP antibody (Supplementary Table S1) to verify successful immunoprecipitation. The remaining immunoprecipitated complexes were incubated in proteinase K digestion buffer (100 mM Tris-HCl, pH 7.5, 50 mM NaCl, 10 mM EDTA, and 0.5% SDS) containing 0.2 mg/mL proteinase K for 30 min at 55 °C. RNA was extracted using TRIzol reagent (Invitrogen) according to the manufacturer’s instructions, precipitated with glycogen and isopropanol, washed with 75% ethanol, and resuspended in RNase-free water. First-strand cDNA was synthesized using random hexamer primers and M-MLV reverse transcriptase (Promega). The enrichment of Hk2 mRNA and control transcripts in FMRP immunoprecipitates was quantified by RIP-qPCR using SYBR Green Master Mix (Bio-Rad). Primer sequences are provided in Supplementary Table S2.

To validate the specificity of the RIP assay, Map1b and Dlg4, two established FMRP-associated transcripts, were included as positive controls, whereas Gapdh mRNA and an intergenic RNA region were included as negative controls. Relative RNA enrichment was normalized to the corresponding input RNA and is presented as fold enrichment relative to the matched IgG immunoprecipitate (IgG = 1). All RIP experiments were performed using at least three independent biological replicates.

### ELISA assay

HK2 protein levels were measured using a commercial ELISA kit (MBS2705063, MyBioSource) according to the manufacturer’s instructions. Absorbance at 450 nm was measured using a microplate spectrophotometer (Epoch, BioTek, VT, USA). Protein concentrations were calculated based on the standard curve generated from the provided HK2 standards. All samples were analyzed in triplicate.

### Morris water maze

The Morris water maze test was performed to evaluate spatial learning and memory. The circular pool was divided into four equal quadrants, and visual cues were positioned around the testing room. Mice underwent training for 7 consecutive days with four trials per day to locate a submerged platform. Each trial lasted a maximum of 60 s. Mice that failed to find the platform within 60 s were guided to the platform and allowed to remain there for 15 s. Escape latency was recorded for each trial. Probe tests were performed 24 h after the final training session by removing the platform, and the time spent in the target quadrant and the number of platform crossings were recorded. Reversal learning was assessed by relocating the platform to the opposite quadrant, followed by additional training and probe testing. Swimming speed was analyzed to exclude potential motor deficits.

### Western blotting

Cells or hippocampal tissues were lysed in lysis buffer supplemented with a protease inhibitor cocktail, and lysates were clarified by centrifugation. Protein concentrations were determined using a BCA protein assay kit. Equal amounts of protein were mixed with loading buffer, boiled, and separated by 10%–12% SDS-PAGE before being transferred onto polyvinylidene fluoride (PVDF) membranes. Membranes were blocked with 5% non-fat milk for 2 h and incubated overnight at 4 °C with primary antibodies, followed by incubation with horseradish peroxidase (HRP)-conjugated secondary antibodies for 2 h at room temperature. The information of all primary antibodies, including antibody suppliers and catalog numbers, is provided in Supplementary Table S1. Immunoreactive bands were visualized using enhanced chemiluminescence (ECL) reagents and quantified using GeneGnome XRQ imaging system and ImageJ software. Target protein levels were normalized to appropriate loading controls. All experiments were independently repeated at least three times.

### Statistical analysis

Sample sizes and biological replicates for each experiment are indicated in the corresponding figure legends. Statistical analyses were performed using GraphPad Prism 10. Data are in the format of the mean ± standard error of the mean (SEM). The Shapiro-Wilk test served for normality assessment. Two-group comparison was analyzed with two-tailed Student’s t-tests when normality criteria were met. Multiple-group comparisons were carried out using one-way ANOVA followed by the appropriate *post hoc* tests where applicable. The specific statistical tests used for each experiment are indicated in the corresponding figure legends. A value of *P* < 0.05 was considered statistically significant. Statistical significance was indicated as *P < 0.05, **P < 0.01, ***P < 0.001.

## Results

### HK2 expression is increased in hippocampal neurons of *Fmr1* ^*-/Y*^ mice

**FIGURE 1:**
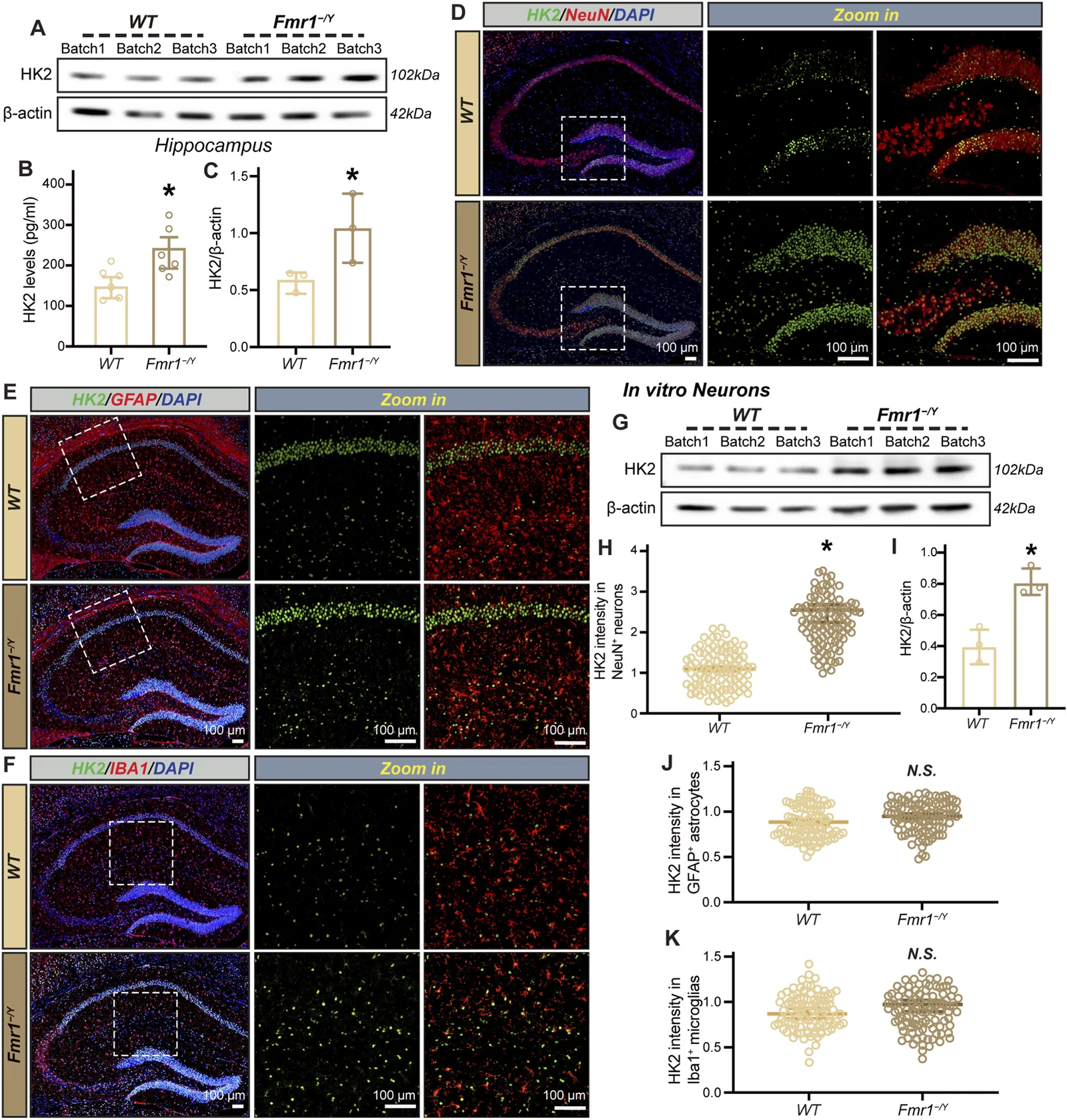
HK2 expression is elevated in hippocampal neurons of *Fmr1* ^*−/Y*^ mice **(A–C)** Western blot and ELISA analyses of HK2 expression in hippocampal lysates from wild-type (WT) and *Fmr1* ^*−/Y*^ mice. **(A)** Representative Western blot image; **(B)** ELISA quantification of HK2 protein levels; **(C)** Densitometric quantification of Western blot results (n = 3 mice per group). **(D–F)** Representative immunofluorescence images showing HK2 co-staining with markers for neurons (NeuN, D), astrocytes (GFAP, E), and microglia (Iba1, F) in the hippocampus of WT and *Fmr1* ^*−/Y*^ mice. **(G, I)** Western blot analysis of HK2 expression in primary hippocampal neurons isolated from WT and *Fmr1* ^*−/Y*^ mice. **(G)** Representative Western blot images; **(I)** Quantification of HK2 protein levels (n = 3 independent cultures per group). **(H, J, K)** Quantification of HK2 fluorescence intensity in NeuN-positive neurons **(H)**, GFAP-positive astrocytes **(J)**, and Iba1-positive microglia **(K)**. Data are presented as mean ± SEM. Statistical comparisons were performed using an unpaired two-tailed Student’s t-test. A value of *P* < 0.05 was considered statistically significant. *P < 0.05 *versus* WT; N.S., not significant. Scale bar = 100 μm.

To investigate whether glycolytic metabolism regulation is altered in FXS, we first examined HK2 expression in the hippocampus of *Fmr1* ^*−/Y*^ mice. Western blot and ELISA analyses demonstrated significantly increased HK2 protein levels in the hippocampus of *Fmr1* ^*−/Y*^ mice compared with WT controls (Figures 1A–C). To determine the cellular specificity of HK2 upregulation, immunofluorescence co-staining was performed using markers for neurons (NeuN), astrocytes (GFAP), and microglia (Iba1). Although HK2 immunoreactivity was detected in multiple cell types, HK2 signal intensity was selectively increased in hippocampal neurons of *Fmr1* ^*−/Y*^ mice (Figures 1D,H). Astrocytes and microglia showed no significant differences in HK2 signal intensity between *Fmr1* ^*−/Y*^ and WT mice (Figures 1E,F,J,K). Western blot analysis of primary hippocampal neuron cultures further confirmed increased HK2 expression in *Fmr1* ^*−/Y*^ neurons compared with WT controls (Figures 1G,I). These results indicate that HK2 upregulation in *Fmr1* ^*−/Y*^ mice occurs predominantly in hippocampal neurons, suggesting that neuronal HK2 dysregulation may represent a potential metabolic alteration associated with synaptic dysfunction in FXS.

### HK2 suppression enhances neurite outgrowth and synapse formation

**FIGURE 2:**
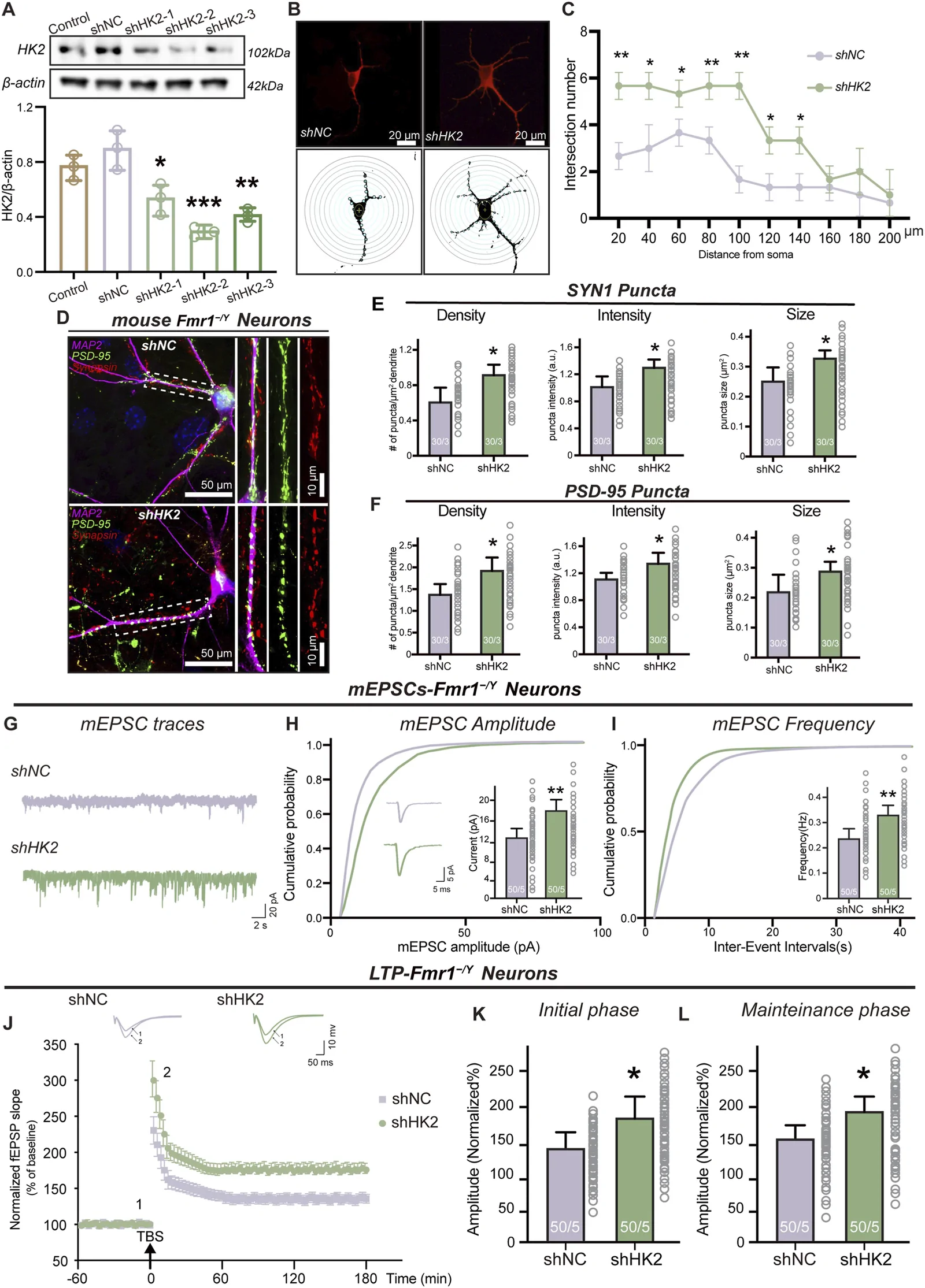
HK2 knockdown improves neuronal morphology, synaptic structure and synaptic plasticity in *Fmr1* ^*−/Y*^ neurons. **(A)** Western blot analysis of HK2 expression in primary hippocampal neurons from *Fmr1* ^*−/Y*^ mice transduced with shNC or shHK2 lentivirus (n = 3 independent cultures per group). **(B)** Representative images of neurons treated with shNC or shHK2, with superimposed concentric circles used for Sholl analysis. **(C)** Quantification of dendritic intersections (15 neurons per group from three independent replicates). **(D)** Representative immunofluorescence images of neurons stained for presynaptic synapsin I (SYN1), postsynaptic marker PSD95, and neuronal marker MAP2. Enlarged dendrite segments are shown in the right panels. **(E, F)** Quantification of puncta density, intensity, and size for SYN1 **(E)** and PSD95 **(F)**. **(G)** Representative mEPSC traces recorded from shNC- and shHK2-treated neurons at day 14. **(H, I)** Cumulative probability distributions of mEPSC amplitude **(H)** and inter-event intervals **(I)**. Insets show averaged mEPSC waveforms (left) and quantification of mEPSC amplitude and frequency (right) Data were obtained from 50 recordings from 10 mice per group. **(J)** Long-term potentiation (LTP) was recorded in the dentate gyrus of *Fmr1* ^*−/Y*^ mice 2 weeks after stereotaxic injection of shNC or shHK2 lentivirus at 2-month age. Upper panels show representative fEPSP traces at indicated time points. The lower panel shows the time course of normalized fEPSP slopes following theta-burst stimulation (TBS). **(K, L)** Quantification of the induction **(K)** and maintenance **(L)** phases of LTP (Data were obtained from 50 recordings from 10 mice per group). Data are presented as mean ± SEM. Statistical analyses were performed using an unpaired two-tailed Student’s t-test unless otherwise indicated. Sholl analysis **(C)** was analyzed using one-way ANOVA followed by Tukey’s multiple-comparison test. *p < 0.05; **p < 0.01; ***p < 0.001 *versus* shNC.

Given the elevated expression of HK2 in *Fmr1* ^*−/Y*^ neurons, we next investigated whether its aberrant upregulation is associated with neuronal morphological and synaptic abnormalities in FXS. To investigate the functional role of HK2 in FXS neurons, cultured hippocampal neurons isolated from *Fmr1* ^*−/Y*^ mice were transduced at DIV5 with lentiviral vectors expressing HK2-targeting shRNAs. Western blot analysis confirmed that all three HK2-targeting shRNAs (shHK2-1, shHK2-2 and shHK2-3) effectively reduced HK2 expression. Among them, shHK2-2 showed the most efficient reduction of HK2 expression and was therefore selected for subsequent experiments (Figure 2A). We then assessed neuronal morphology at DIV14 using MAP2 immunostaining (Figure 2B). Sholl analysis revealed that HK2 knockdown increased dendritic complexity and branching, as indicated by increased dendritic intersections across multiple radial distances from the Soma compared to the shNC control group (Figure 2C). To determine whether these morphological changes were accompanied by alterations in synaptic structures, we quantified the density, fluorescence intensity, and size of pre- and post-synaptic puncta via immunostaining for SYN1 and PSD-95 (Figure 2D). Notably, depletion of HK2 significantly increased the density, intensity, and puncta size of both SYN1 (Figure 2E) and PSD-95 (Figure 2F), suggesting improved synaptic structural organization in *Fmr1* ^*−/Y*^ neurons.

To determine whether these structural changes were accompanied by functional alterations in synaptic transmission, we next performed whole-cell patch-clamp recordings of miniature excitatory postsynaptic currents (mEPSCs) (Figure 2G). Both the amplitude (Figure 2H) and frequency (Figure 2I) of mEPSCs were significantly increased in shHK2-treated neurons, as evidenced by shifts in cumulative probability curves and summarized data, suggesting enhanced excitatory synaptic transmission in *Fmr1* ^*−/Y*^ neurons. Given these alterations in basal synaptic transmission, we further evaluated long-term potentiation (LTP) to assess synaptic plasticity (Figure 2J). HK2-knockdown neurons exhibited enhanced LTP, characterized by increased normalized fEPSP slopes during both the induction phase (Figure 2K) and maintenance phase (Figure 2L). Taken together, these findings indicate that HK2 knockdown improves neuronal morphology, enhances excitatory synaptic transmission, and enhances synaptic plasticity in *Fmr1* ^*−/Y*^ neurons, suggesting that aberrant HK2 upregulation is involved in synaptic dysfunction in FXS.

### HK2 is associated with abnormal H3K18 lactylation in FXS neurons

**FIGURE 3:**
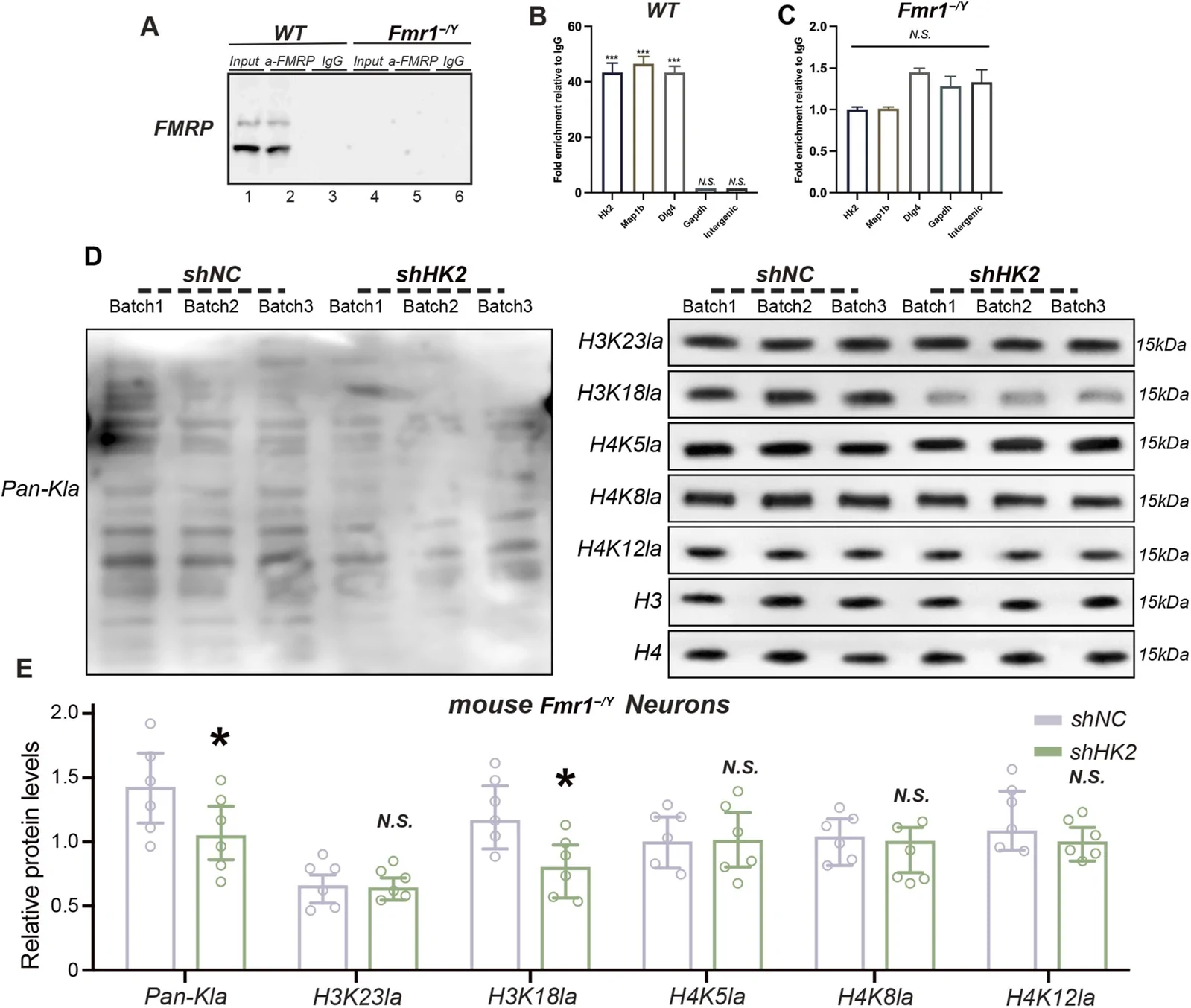
FMRP associates with HK2 mRNA, and HK2 alters H3K18 lactylation in FXS neurons. **(A)** Western blot confirming successful immunoprecipitation of FMRP protein using an anti-FMRP antibody (IP: FMRP), but not control IgG, in WT samples; no FMRP was detected in *Fmr1* ^*−/Y*^ samples as expected. **(B, C)** RIP-qPCR analysis of *Hk2* mRNA and control transcripts in anti-FMRP immunoprecipitates from WT and *Fmr1* ^*−/Y*^ hippocampi. In WT samples, *Hk2* mRNA and the established FMRP target transcripts *Map1b* and *Dlg4* were significantly enriched in anti-FMRP immunoprecipitates compared with the corresponding IgG immunoprecipitates, with enrichment expressed as fold enrichment relative to IgG (IgG = 1), whereas *Gapdh* mRNA and an intergenic RNA region showed no significant enrichment. In *Fmr1* ^*−/Y*^ hippocampi, no significant enrichment of the tested transcripts relative to IgG controls was detected, confirming the specificity of the RIP assay. **(D)** Representative immunoblots showing pan-lactylation (Pan-Kla) and site-specific histone lactylation marks (H3K23la, H3K18la, H4K5la, H4K8la, and H4K12la) in hippocampi from *Fmr1* ^*−/Y*^ mice treated with shNC or shHK2. **(E)** Quantification of Pan-Kla and site-specific histone lactylation levels shown in **(D)**. Data are presented as mean ± SEM (n = 6 mice per group). Statistical comparisons were performed using an unpaired two-tailed Student’s t-test. *p < 0.05 *versus* shNC; N.S., not significant.

Given the established role of FMRP as an RNA-binding protein involved in post-transcriptional regulation, we next investigated whether *Hk2* mRNA is associated with FMRP. RNA immunoprecipitation (RIP) assays were performed using hippocampal tissues from WT and *Fmr1* ^*−/Y*^ mice. Successful immunoprecipitation of FMRP was first confirmed by Western blot analysis, which showed robust enrichment of FMRP in anti-FMRP immunoprecipitates from WT samples but not from *Fmr1* ^*−/Y*^ mice or IgG controls (Figure 3A). RIP-qPCR analysis further demonstrated significant enrichment of *Hk2* mRNA in anti-FMRP immunoprecipitates relative to the corresponding IgG controls in WT samples, whereas no enrichment was detected in *Fmr1* ^*−/Y*^ samples (Figures 3B,C). The established FMRP-associated transcripts *Map1b* and *Dlg4* showed similar enrichment, whereas *Gapdh* mRNA and an intergenic RNA region were not enriched, confirming the specificity of the RIP assay. These findings indicate that *Hk2* mRNA is associated with FMRP, suggesting that HK2 may represent an FMRP-associated transcript; however, additional studies are required to determine whether FMRP directly regulates HK2 translation.

Since HK2 is a key regulator of glycolytic flux and lactate production, we next investigated whether HK2 dysregulation affects histone lactylation, a metabolite-derived epigenetic modification. Western blot analysis revealed that HK2 knockdown significantly reduced global histone lactylation levels in the hippocampus of *Fmr1* ^*−/Y*^ mice. Notably, among the lactylation marks examined, H3K18la exhibited the most pronounced reduction following HK2 suppression, whereas H3K23la, H4K5la, H4K8la, and H4K12la showed no significant changes (Figures 3D,E). These findings indicate that H3K18la showed the most prominent reduction among the examined lactylation marks following HK2 suppression. Together, these findings suggest that HK2 dysregulation is associated with altered H3K18la levels and support a potential association among FMRP deficiency, HK2 dysregulation, and altered histone lactylation in FXS neurons.

### Pharmacological elevation of H3K18la modulates the effects of HK2 inhibition

**FIGURE 4:**
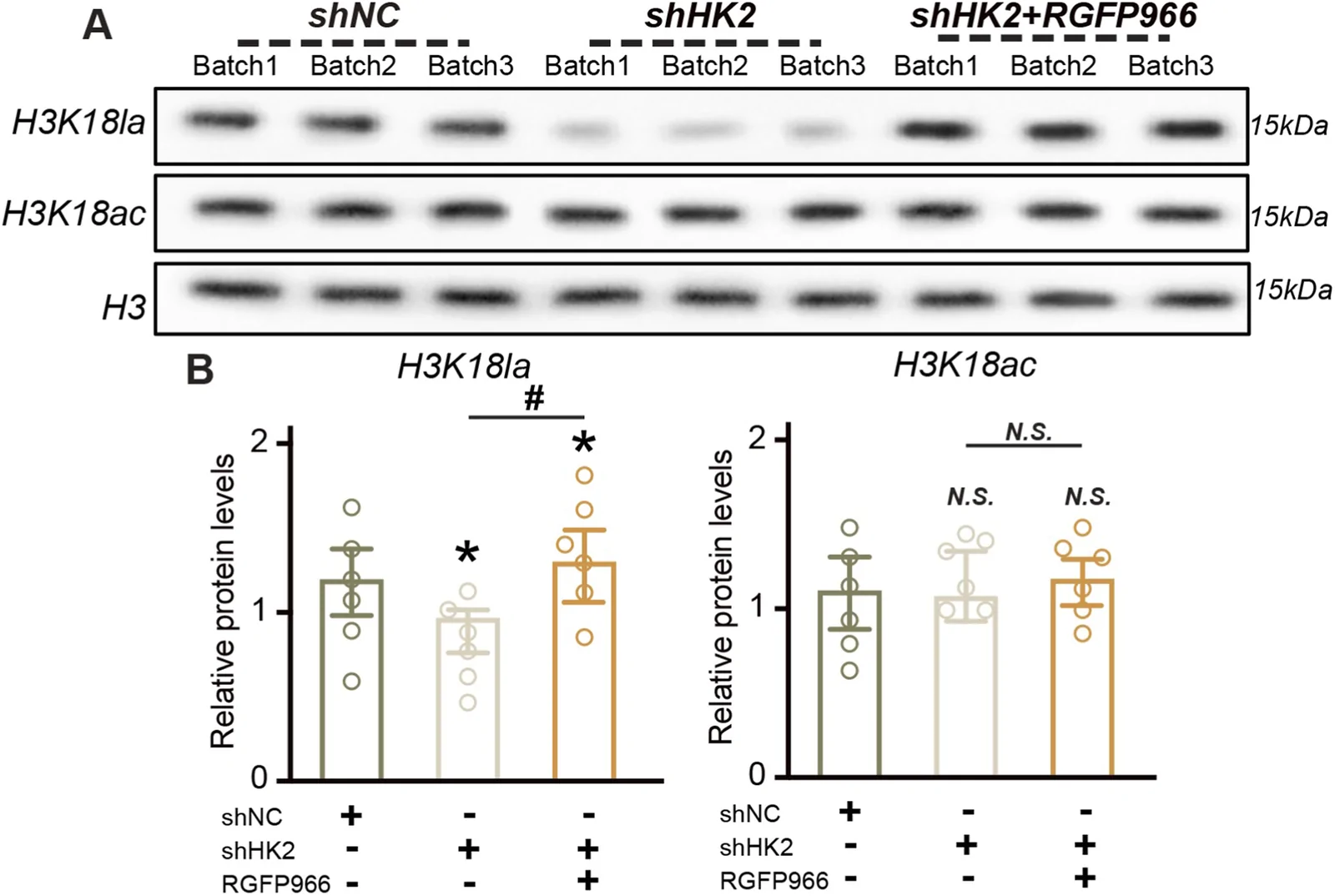
HK2 knockdown alters H3K18 lactylation levels in *Fmr1* ^*−/Y*^ mouse neurons. **(A)** Western blot analysis of H3K18 lactylation (H3K18la) and H3K18 acetylation (H3K18ac) in hippocampi from 3-month-old *Fmr1* ^*−/Y*^ mice treated with shNC, shHK2, or shHK2 plus HDAC3 inhibitor RGFP966. **(B)** Quantification of H3K18la and H3K18ac levels from **(A)**. Data are mean ± SEM. Statistical comparisons were performed using one-way ANOVA followed by Tukey’s multiple-comparison test. *p < 0.05 *versus* shNC, ^#^ *p* < 0.05 *versus* shHK2; N.S., not significant.

Histone lactylation is dynamically regulated by the coordinated actions of lactylation “writers” and “erasers.” Recent studies have suggested that HDAC3 may function as a regulator of histone lactylation dynamics. To examine whether modulation of histone lactylation influences HK2-associated changes in H3K18la, we treated HK2-deficient *Fmr1* ^*−/Y*^ mice with RGFP966, a selective HDAC3 inhibitor. Western blot analysis revealed that RGFP966 treatment increased hippocampal H3K18la levels compared with HK2 knockdown alone, whereas H3K18ac levels remained largely unchanged (Figures 4A,B). These findings suggest that HDAC3 inhibition preferentially affects H3K18la levels under these experimental conditions and further support a potential link between HK2 expression and H3K18la regulation.

To further examine the functional relationship between HK2-associated H3K18la changes and neuronal phenotypes, RGFP966 was used to pharmacologically modulate H3K18la levels in HK2-deficient *Fmr1* ^*−/Y*^ mice. This experimental strategy allowed us to examine whether pharmacological elevation of H3K18la influences the effects of HK2 knockdown on neuronal morphology and synaptic function.

### H3K18 lactylation contributes to the synaptic effects of HK2 suppression

**FIGURE 5:**
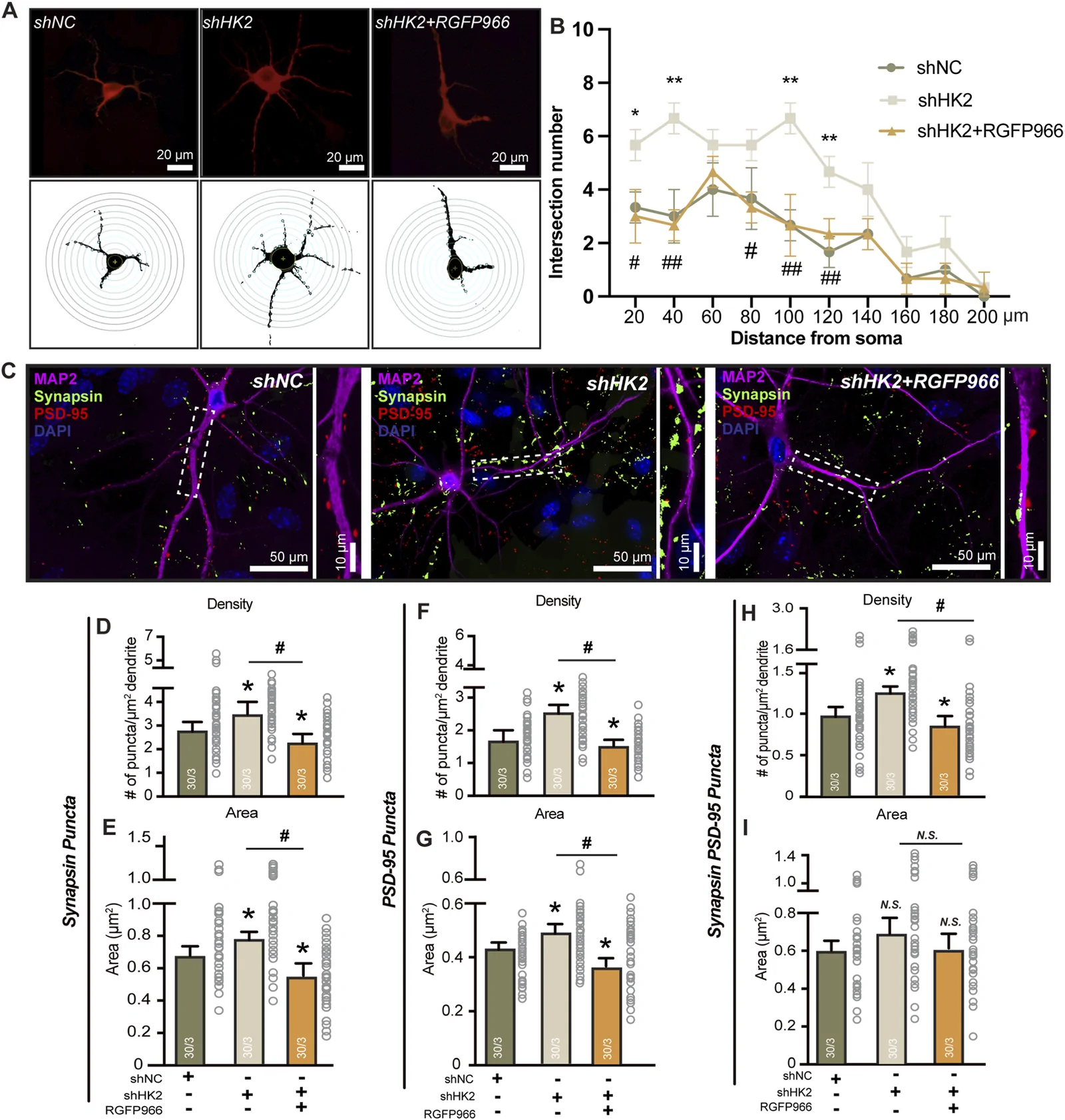
H3K18la modulation affects HK2 knockdown-induced changes in neuronal morphology and synapse structure. **(A)** Representative MAP2 immunostaining images of *Fmr1* ^*−/Y*^ neurons transduced with shNC, shHK2, or shHK2 plus RGFP966. Lower panels: corresponding Sholl analyses with concentric circles. **(B)** Sholl analysis quantifying dendritic intersections as a function of distance from Soma under indicated treatments (15 neurons per group from three independent replicates). **(C)** Representative images of neurons stained for synapsin I, PSD95, and MAP2 after the indicated treatments. Enlarged dendrite segments are shown in the right panels. **(D, E)** Quantification of SYN1-positive puncta density **(D)** and puncta area **(E)** in dendrites. **(F, G)** Quantification of PSD95-positive puncta density **(F)** and puncta area **(G)**. **(H, I)** Quantification of colocalized SYN1/PSD95 puncta density **(H)** and area **(I)**, representing synaptic structural organization. Data are presented as means ± SEM. The number of analyzed cells and independent cultures is indicated within the bars. Statistical analyses were performed using one-way ANOVA followed by Tukey’s multiple-comparison test. *p < 0.05; **p < 0.01 *versus* shNC; ^#^ *p* < 0.05 vs. shHK2; N.S. not significant.

To further determine whether H3K18la contributes to HK2-dependent synaptic phenotypes, immature hippocampal neurons were transduced with shHK2 at DIV5 and cultured until DIV14. RGFP966 was administered 3 days prior to analysis. Neuronal morphology was analyzed at DIV14 by MAP2 immunostaining. HDAC3 inhibition attenuated the enhancement of neurite outgrowth and dendritic complexity observed following HK2 knockdown, as demonstrated by decreased total dendritic branching (Figure 5A) and reduced Sholl intersections (Figure 5B). Consistent with these morphological findings, RGFP966 treatment also reduced the synaptic structural improvements observed following HK2 suppression (Figure 5C). Quantification analysis revealed significant reductions in both the densities and average areas of synapsin I- and PSD95-positive puncta (Figures 5D–G). Similarly, the density of colocalized synapsin I/PSD95 puncta was significantly decreased, although its mean area remained unaltered (Figures 5H,I). These results suggest that HDAC3 inhibition-mediated elevation of H3K18la may contribute to the attenuation of HK2 knockdown-induced neuronal improvements.

**FIGURE 6:**
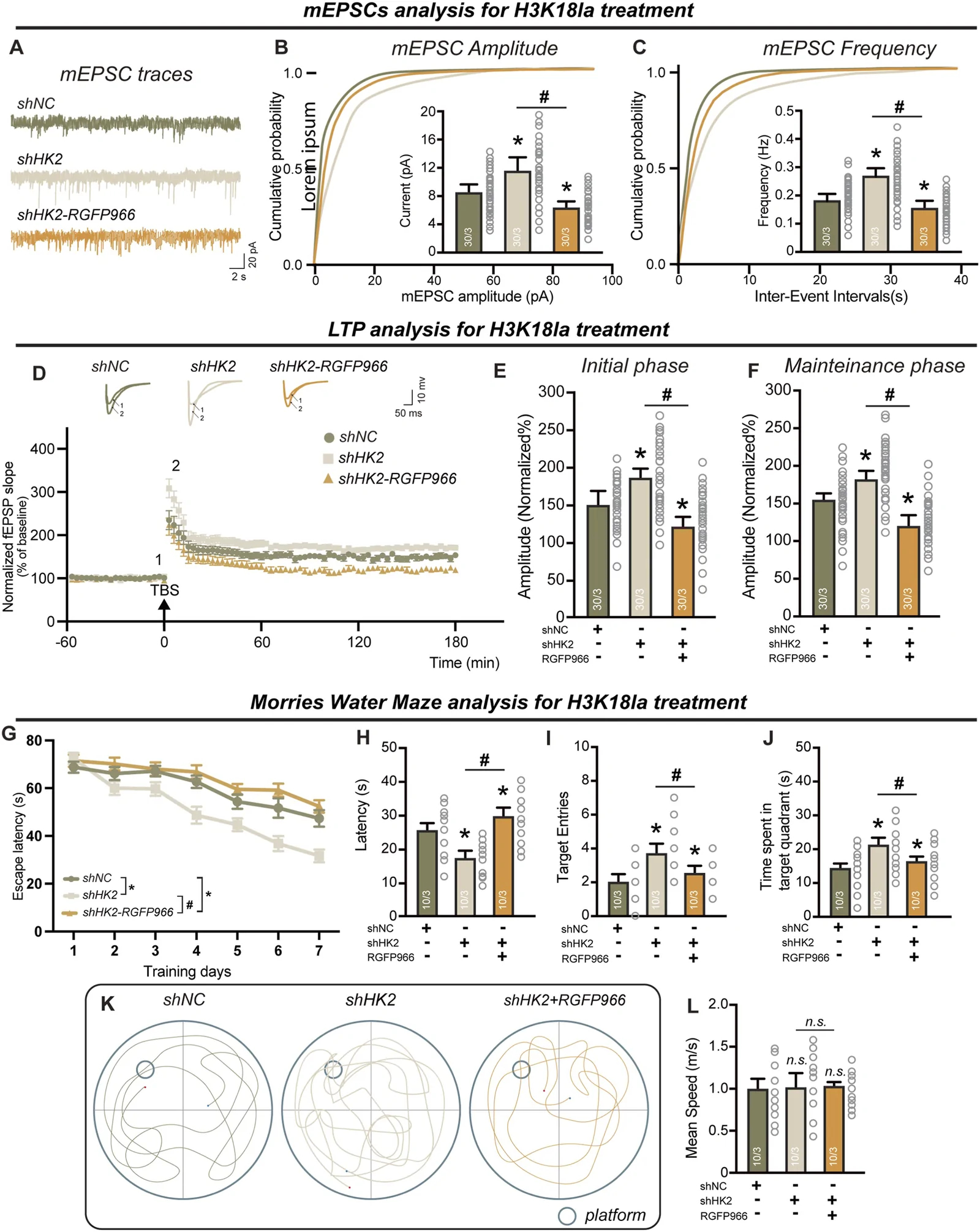
H3K18la modification affects synaptic plasticity, spatial learning, and memory following HK2 knockdown in *Fmr1* ^*−/Y*^ mice. **(A)** Representative mEPSC traces recorded from primary hippocampal neurons at DIV14 following treatment with shNC, shHK2, or shHK2 + RGFP966. **(B, C)** Cumulative probability plots showing amplitudes **(B)** and inter-event intervals **(C)** of mEPSCs. Insets show mean mEPSC amplitude **(B)** and frequency **(C)**. **(D)** LTP recordings from hippocampal DG region. Representative fEPSP traces at indicated time points (top panel) and fEPSP amplitude during a 180-min recording period (bottom panel). **(E, F)** Quantification of LTP induction **(E)** and maintenance **(F)** phases (30 responses averaged per mouse; n = 10 mice per group). **(G)** Escape latency during Morris water maze acquisition training (n = 10 mice per group). **(H–J)** Latency to first enter the target zone **(H)**, number of target zone entries **(I)**, and time spent in the target quadrant **(J)** during the probe trial (n = 10 mice per group). **(K)** Representative swim paths of mice during the Morris water maze probe trial. **(L)** Mean swimming speed during the probe trial (n = 10 mice per group). Data are presented as means ± SEM. Statistical comparisons were performed using one-way ANOVA followed by Tukey’s multiple-comparison test ^*^ *p* < 0.05 *versus* shNC; ^#^ *p* < 0.05 *versus* shHK2; N.S., not significant.

Electrophysiological analyses further demonstrated that HDAC3 inhibition attenuated the increases in mEPSC frequency and amplitude observed following HK2 knockdown, as indicated by both shifted cumulative probability curves and altered mean values (Figures 6A–C). Moreover, although HK2 suppression enhanced both the induction and maintenance phases of hippocampal long-term potentiation (LTP), concurrent HDAC3 inhibition attenuated these HK2 knockdown-induced improvements (Figures 6D–F). Collectively, these findings indicate that H3K18la is involved, at least in part, in the neuronal and synaptic improvements associated with HK2 knockdown.

### H3K18 lactylation contributes to the cognitive effects associated with HK2 knockdown in *Fmr1* ^*−/Y*^ mice

To determine whether the HK2–H3K18la regulatory axis influences cognitive function *in vivo*, behavioral consequences were further evaluated using the Morris water maze. Compared with *Fmr1* ^*−/Y*^ mice treated with shHK2 alone, mice receiving combined shHK2 and RGFP966 treatment showed attenuated improvements in spatial learning and memory. This reduction in cognitive improvement was reflected by increased escape latency during the 7-day training phase (Figure 6G). In the probe test, the combined treatment group showed increased latency to reach the target location (Figure 6H), fewer platform crossings (Figure 6I), and reduced time spent in the target quadrant (Figures 6J,K). No significant differences in swimming speed were observed among groups (Figure 6L), suggesting that the behavioral differences were not attributable to alterations in locomotor ability. These findings suggest that H3K18la may contribute to the cognitive effects associated with HK2 suppression.

## Discussion

HK2 is a key glycolytic enzyme that catalyzes the first committed step of glucose metabolism and plays an important role in regulating cellular energy utilization (Thomas et al., 2022). Although HK2 has been extensively studied in cancer metabolism, where its upregulation promotes glycolytic reprogramming and tumor progression (DeWaal et al., 2018), increasing evidence indicates that HK2 is also highly expressed in the nervous system and contributes to neuronal energy metabolism and synaptic function (Li et al., 2018). Aberrant HK2 expression has been implicated in multiple neurological disorders, including Alzheimer’s disease, Parkinson’s disease, and ischemic brain injury (Li et al., 2022). In the present study, we found that HK2 expression was selectively elevated in hippocampal neurons of *Fmr1* ^*−/Y*^ mice. Our RIP analysis revealed an association between FMRP and HK2 mRNA, suggesting that HK2 may be regulated by FMRP-associated post-transcriptional mechanisms. Consistent with this possibility, HK2 mRNA expression remained unchanged in both the hippocampus and primary hippocampal neurons from *Fmr1* ^*−/Y*^ mice (Supplementary Figure S1), indicating that the increased HK2 protein expression is unlikely to be explained by altered transcript abundance. However, RIP analysis alone is insufficient to establish a translational mechanism. Future studies employing polysome profiling or nascent protein labeling will therefore be required to determine whether FMRP influences HK2 translational efficiency. Functional analyses further revealed that HK2 suppression enhances synaptic structural integrity, and improves synaptic transmission, hippocampal synaptic plasticity, and cognitive performance. Together, these findings identify HK2 dysregulation as a potential metabolic contributor to synaptic dysfunction in FXS and support a previously underappreciated role for metabolic dysregulation in the pathogenesis of this disorder.

Beyond its neuronal functions, HK2 has also been implicated in metabolic reprogramming and inflammatory signaling within the central nervous system, particularly in microglia (Hu et al., 2022; Fang et al., 2023). Activated microglia undergo a metabolic shift from oxidative phosphorylation to glycolysis, and HK2 upregulation has been associated with inflammatory activation through enhanced glycolytic metabolism and ROS production (Cantando et al., 2024; Bennett et al., 2020). Previous studies have shown that HK2 inhibition reduces microglial activation, neuroinflammation, and neuronal injury in several neurodegenerative and neuroinflammatory disorders (Leng et al., 2022; Li et al., 2022; Eming et al., 2021; Liu et al., 2021; Huang et al., 2024; Codocedo et al., 2024). In contrast, our immunofluorescence analyses revealed no significant changes in HK2 expression in hippocampal microglia from *Fmr1* ^*−/Y*^ mice, whereas a marked increase was observed in neurons. These findings suggest that HK2 dysregulation in FXS exhibits cell-type specificity, with hippocampal neurons representing the predominant cellular population exhibiting HK2 dysregulation in this model. Nevertheless, given the region- and disease-stage-dependent functions of microglia, future studies should determine whether HK2 contributes to microglia–neuron interactions in other brain regions or disease stages of FXS.

A key finding of this study is the identification of histone lactylation as a mechanistic link between HK2-mediated metabolic alterations and synaptic dysfunction in FXS. Increasing evidence supports a close interplay between cellular energy metabolism and epigenetic regulation. Histone lactylation, a recently described post-translational modification derived from lactate produced during glycolysis, has emerged as an important modulator of chromatin structure and gene transcription (M et al., 2024; Sui et al., 2025; Lu et al., 2024). Lactate can serve as a substrate for p300-mediated lysine lactylation, notably at histone H3 lysine 18 (H3K18la), thereby directly linking cellular metabolism to transcriptional regulation. Previous studies have implicated histone lactylation in regulating inflammatory and metabolic gene expression in neurological disorders (Pan et al., 2022). In the present study, elevated HK2 expression in *Fmr1* ^*−/Y*^ mice was associated with increased H3K18la levels, whereas HK2 knockdown preferentially reduced H3K18la while leaving H3K18 acetylation largely unchanged. Notably, among the lactylation marks examined, H3K18la exhibited the most prominent response to HK2 suppression, whereas H3K23la, H4K5la, H4K8la, and H4K12la remained largely unchanged. Furthermore, pharmacological modulation of H3K18la through HDAC3 inhibition significantly attenuated the beneficial effects of HK2 knockdown on neuronal morphology, synaptic transmission, synaptic plasticity, and cognitive performance. Together, these findings suggest that H3K18la may represent an epigenetic mechanism associated with HK2-related neuronal phenotypes and highlight a potential HK2–H3K18la metabolic–epigenetic axis contributing to synaptic dysfunction in FXS.

Despite these findings, several important mechanistic questions remain to be addressed. Although our results support a regulatory role for HK2 in H3K18 lactylation, direct measurement of intracellular lactate levels will be important to further define the metabolic mechanisms linking HK2 activity to histone lactylation. Future studies incorporating functional metabolic analyses to directly assess glycolytic activity, together with ^13C-glucose isotope tracing approaches and lactate rescue experiments may provide additional insight into the metabolic flux underlying this process and help clarify the contribution of lactate to the downstream effects of HK2. In addition, although RGFP966 treatment increased H3K18la levels in the present study, we cannot completely exclude the contribution of additional HDAC3-dependent epigenetic mechanisms to the observed phenotypes. Moreover, while H3K18la exhibited the most prominent HK2-dependent change among the histone lactylation sites examined, additional histone lactylation sites and non-histone lactylated proteins may also contribute to the pathogenesis of FXS. Future comprehensive lactylome analyses will therefore be important for defining the broader landscape of protein lactylation in FXS. Finally, the downstream transcriptional programs through which H3K18la contributes to synaptic dysfunction remain largely unknown. Future studies integrating transcriptomic and epigenomic approaches, such as RNA-seq and CUT&Tag, will facilitate the identification of genes and regulatory networks associated with H3K18la alterations.

In summary, the present study identifies HK2 dysregulation as a metabolic alteration associated with synaptic dysfunction in FXS and highlights H3K18 lactylation as a potential epigenetic mechanism associated with HK2-related neuronal phenotypes. Our findings support a model in which FMRP deficiency is associated with increased HK2 expression and altered metabolic–epigenetic regulation, which may contribute to impairments in synaptic structure, synaptic plasticity, and cognitive function. By identifying an association between HK2 dysregulation and H3K18la alterations, this study expands the understanding of metabolic–epigenetic changes in FXS and highlights the importance of metabolic–epigenetic interactions in neurodevelopmental disorders. These findings suggest that HK2-associated metabolic pathways and their epigenetic consequences may represent potential targets for future therapeutic exploration in FXS and related neurodevelopmental conditions.

## Data Availability

The raw data supporting the conclusions of this article will be made available by the authors, without undue reservation.
