# Supplementary material for: HK2 drives synaptic dysfunction in fragile X syndrome via epigenetic regulation of H3K18 lactylation
Source: Front Pharmacol. 2026 Aug 31;17:1903604. doi: 10.3389/fphar.2026.1903604 (PMC13572682; doi:10.3389/fphar.2026.1903604)
Supplement: Supplementary file 1 [file Supplementaryfile1.docx]

## **Supplementary Methods**

### Quantitative real-time PCR

Total RNA was extracted from **hippocampal tissues** or **primary hippocampal neurons** using TRIzol reagent (Invitrogen, Carlsbad, CA, USA) according to the manufacturer's instructions. RNA concentration and purity were determined using a NanoDrop spectrophotometer (Thermo Fisher Scientific). Equal amounts of total RNA (1 μg) were reverse-transcribed into first-strand cDNA using the PrimeScript™ RT Reagent Kit with gDNA Eraser (Takara, Dalian, China) following the manufacturer's protocol.

Quantitative real-time PCR (qRT-PCR) was performed using TB Green® Premix Ex Taq™ II (Takara) on a QuantStudio™ 5 Real-Time PCR System (Applied Biosystems, Foster City, CA, USA). The PCR cycling conditions were as follows: initial denaturation at 95°C for 30 s, followed by 40 cycles of 95°C for 5 s and 60°C for 30 s. A melting curve analysis was performed after amplification to confirm the specificity of the PCR products.

Relative **Hk2** mRNA expression was normalized to **Gapdh** and calculated using the **2^−ΔΔCt** method. Primer sequences used for qRT-PCR are listed in ****Supplementary Table S2****. Each experimental group included **three independent biological replicates (n = 3)**, and all reactions were performed in technical triplicate.
