# Supplementary material for: HK2 drives synaptic dysfunction in fragile X syndrome via epigenetic regulation of H3K18 lactylation
Source: Front Pharmacol. 2026 Aug 31;17:1903604. doi: 10.3389/fphar.2026.1903604 (PMC13572682; doi:10.3389/fphar.2026.1903604)
Supplement: Supplementary file 2 [file Supplementaryfile2.docx]

 Supplementary Fig. S1

Supplementary Fig. S1: (A) Quantitative real-time PCR analysis of Hk2 mRNA expression in the hippocampus of WT and *Fmr1^−/Y^* mice. (B) Quantitative real-time PCR analysis of Hk2 mRNA expression in primary hippocampal neurons derived from WT and *Fmr1^−/Y^* mice. Hk2 mRNA levels were normalized to Gapdh and quantified using the 2^-ΔΔCt method. Data are presented as mean ± SEM (n = 3 biological replicates per group). Statistical analyses were performed using an unpaired two-tailed Student's t-test. N.S., not significant.
