# Supplementary material for: HK2 drives synaptic dysfunction in fragile X syndrome via epigenetic regulation of H3K18 lactylation
Source: Front Pharmacol. 2026 Aug 31;17:1903604. doi: 10.3389/fphar.2026.1903604 (PMC13572682; doi:10.3389/fphar.2026.1903604)
Supplement: Supplementary file 3 [file Table1.docx]

***Table 1. REAGENT or RESOURCE***

| *REAGENT or RESOURCE* | | *SOURCE* | *IDENTIFIER* |  |
| --- | --- | --- | --- | --- |
| Rabbit monoclonal anti-HK2 | | Abcam | Cat# ab209847 |  |
| Mouse monoclonal anti-NeuN | | Abcam | Cat# ab104224 |  |
| Mouse monoclonal anti-GFAP | | Millipore | Cat# MAB360 |  |
| Mouse monoclonal anti-IBA1 | | Abcam | Cat# ab283319 |  |
| Mouse Polyclonal anti-TUJ1 | | Invitrogen | Cat# **PA5-85639** |  |
| Chicken polyclonal anti-MAP2 | | Encor | RRID: AB_2138173 |  |
| Rabbit monoclonal anti-Synapsin I | | Abcam | Cat# ab254349 |  |
| Mouse monoclonal anti-PSD95 | | Thermo | Cat# MA1-046 |  |
| Rabbit monoclonal anti-FMRP | | Abcam | Cat# ab259335 |  |
| Rabbit polyclonal anti-Pan Kla | | PTM BIO | Cat# PTM-1401 |  |
| Rabbit monoclonal anti-H3 | | PTM BIO | Cat# PTM-1001RM |  |
| Mouse monoclonal anti-H4 | | PTM BIO | Cat# PTM-1009 |  |
| Rabbit monoclonal anti-H3K18la | | PTM BIO | Cat# PTM-1406RM |  |
| Rabbit polyclonal anti-H3K23la | | PTM BIO | Cat# PTM-1413 |  |
| Rabbit monoclonal anti-H4K5la | | PTM BIO | Cat# PTM-1407RM |  |
| Rabbit monoclonal anti-H4K8la | | PTM BIO | Cat# PTM-1415RM |  |
| Rabbit monoclonal anti-H4K12la | | PTM BIO | Cat# PTM-1411RM |  |
| Rabbit monoclonal anti-H3K18ac | | PTM BIO | Cat# PTM-114RM |  |
| *RIP antibodies* | | | |  |
| Rabbit monoclonal anti-FMRP antibody | | Cell Signaling Technology | Cat# 4317 |  |
| Normal rabbit IgG control | | Millipore | Cat# 12-370 |  |
| *Sequences of shRNAs targeting HK2* | | | |  |
| shRNA | Sequence | | | |
| HK2 | Forward: 5’ CCGGTCCAAAGACATCTCAGACATTGTTCAAGAGACAATGTCTGAGATGTCTTTGGTTTTTG 3’ | | | |
|  | Reverse: 5’ AATTCAAAAACCAAAGACATCTCAGACATTGTCTCTTGAACAATGTCTGAGATGTCTTTGGA 3’ | | | |
| Negative Control | Forward: 5’ CCGGTTTCTCCGAACGTGTCACGTTTCAAGAGAACGTGACACGTTCGGAGAATTTTTG 3’ | | | |
|  | Reverse: 5’ AATTCAAAAATTCTCCGAACGTGTCACGTTCTCTTGAAACGTGACACGTTCGGAGAAA 3’ | | | |
