# Supplementary material for: HK2 drives synaptic dysfunction in fragile X syndrome via epigenetic regulation of H3K18 lactylation
Source: Front Pharmacol. 2026 Aug 31;17:1903604. doi: 10.3389/fphar.2026.1903604 (PMC13572682; doi:10.3389/fphar.2026.1903604)
Supplement: Supplementary file 4 [file Table2.docx]

| Target | Primer Type | Sequence (5' → 3') | Amplicon Size (bp) |
| --- | --- | --- | --- |
| *Hk2* | Forward | 5'-GCC ATC CTC GCT GAC ATT AA-3' | 142 |
| *Hk2* | Reverse | 5'-TCC TCA GCA CTT GGA GCA TT-3' | 142 |
| *Map1b* | Forward | 5'-CAC AGC TCA GAG CCA AAC AA-3' | 118 |
| *Map1b* | Reverse | 5'-TGT TGG TGC TGG GAA GGT AT-3' | 118 |
| *Dlg4* | Forward | 5'-GAG GCA AAG GTA CGT CCA AG-3' | 134 |
| *Dlg4* | Reverse | 5'-GAG TTG ATG CTG GGC AGA GT-3' | 134 |
| *Gapdh* | Forward | 5'-AGG TCG GTG TGA ACG GAT TTG-3' | 123 |
| *Gapdh* | Reverse | 5'-TGT AGA CCA TGT AGT TGA GGT CA-3' | 123 |
| *Intergenic (chr5)* | Forward | 5'-TGC TGA GGA CTG GAG AGA GA-3' | 108 |
| *Intergenic (chr5)* | Reverse | 5'-CTC CAC TCC TGG GAG TGA TT-3' | 108 |
